# The rich somatic life of *Wolbachia*

**DOI:** 10.1002/mbo3.390

**Published:** 2016-07-26

**Authors:** Jose E. Pietri, Heather DeBruhl, William Sullivan

**Affiliations:** ^1^ Department of MolecularCell & Developmental BiologyUniversity of CaliforniaSanta CruzCaliforniaUSA; ^2^ Biological Sciences DepartmentCalifornia Polytechnic State UniversitySan Luis ObispoCaliforniaUSA

**Keywords:** cytoskeleton, endosymbiont, horizontal, insect, invasion, migration, nematode, pathogen resistance, somatic, transfer, virus, *Wolbachia*

## Abstract

*Wolbachia* is an intracellular endosymbiont infecting most arthropod and some filarial nematode species that is vertically transmitted through the maternal lineage. Due to this primary mechanism of transmission, most studies have focused on *Wolbachia* interactions with the host germline. However, over the last decade many studies have emerged highlighting the prominence of *Wolbachia* in somatic tissues, implicating somatic tissue tropism as an important aspect of the life history of this endosymbiont. Here, we review our current understanding of *Wolbachia*–host interactions at both the cellular and organismal level, with a focus on *Wolbachia* in somatic tissues.

## 1 Introduction

*Wolbachia* is an intracellular bacterium found primarily in arthropods and filarial nematodes. In insects, *Wolbachia* is abundant in both the male and female germlines, though it is vertically transmitted exclusively through the female germline. In filarial nematodes, *Wolbachia* is present only in the female germline, facilitating efficient mitochondria‐like maternal transmission. In the majority of hosts, *Wolbachia* exists as an endosymbiont. That is, it maintains a neutral relationship with its host. In most arthropods, this relationship is facultative, whereas in filarial nematodes, *Wolbachia* maintains a fixed obligate relationship with its host. Furthermore, depending on both intrinsic and extrinsic factors, *Wolbachia* can act as a mutualist, commensalist, or pathogen. Because *Wolbachia* is primarily transmitted through the maternal germline, it maintains an extraordinary ability to influence host reproduction to favor proliferation by infected females. *Wolbachia* biology has recently enjoyed increased interest because of two crucial findings: it is a major cause of pathogenicity associated with parasitic filarial nematodes and it has the ability to reduce the titer of dengue virus and other mosquito‐borne human pathogens when infecting the vector species (Eleftherianos, Atri, Accetta, & Castillo, 2013; Taylor, 2003).

*Wolbachia* was first described by Cowdry (1923) and Hertig and Wolbach (1924) as a gram‐negative, intracellular, Rickettsiae‐like bacteria concentrated in the germline and somatic tissues of a broad array of insects and other arthropods. In his 1936 publication, Marshall Hertig honored his mentor Simeon B. Wolbach with the statement, “The name *Wolbachia pipientis* is proposed for the rickettsia of *Culex pipiens*” (Hertig 2013). In the 1970s *Wolbachia* received renewed attention with the classic publication by Yen and Barr demonstrating that a form of reproductive incompatibility (cytoplasmic incompatibility; CI) among mosquito isolates was due to the presence of this maternally inherited, antibiotic curable, rickettsia‐like organism (Yen & Barr, 1973). In infected populations, CI results in infected females maintaining a selective advantage over uninfected females through increased egg hatch rates. Briefly, infected females mated with infected or uninfected males produce viable embryos. In contrast, in unidirectional CI uninfected females mated with infected males produce inviable embryos. Furthermore, in bidirectional CI, mating of males and females infected with different strains of *Wolbachia* also results in the production of inviable embryos (Werren, 1997). Later, additional mechanisms by which *Wolbachia* favor proliferation of infected females in mixed‐infected populations were discovered. These include feminization of genetically male offspring, parthenogenesis by infected females, and male killing of infected males (Louis & Nigro, 1989; Saridaki & Bourtzis, 2010; Werren, Baldo, & Clarke, 2008; Yen & Barr, 1973). Of these mechanisms CI is the most prevalent and well‐studied.

Perhaps because CI operates with the combined effects of *Wolbachia* in the male and female germlines, and *Wolbachia* is transmitted through the latter, much of the work on *Wolbachia* has focused on its interaction with the host germline. However, work over the past decade has reinforced original observations that, in addition to localization to the germline, a conserved feature of *Wolbachia* infection is localization to somatic tissue. Equally significant, these studies have begun to shed light on the functional importance of tissue‐specific somatic localization of *Wolbachia*. Here, we review the current state of knowledge regarding the somatic aspects of *Wolbachia* infection and the functional consequences for both host and endosymbiont.

## 2 An Overview of Distribution in Somatic Tissues

**Figure 1:**
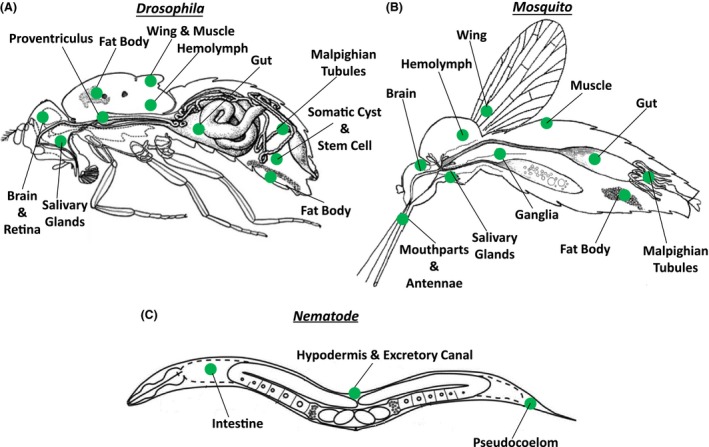
*Wolbachia* distribution in somatic tissues**.** *Wolbachia* has been detected by PCR and fluorescent cytology in various somatic tissues of numerous (A) fly, (B) mosquito, and (C) filarial nematode species, as indicated in green

In the original description of *Wolbachia*, the authors describe not only a concentration of “rodlike organisms in the reproductive tissue but also in the somatic tissue” (Hertig & Wolbach, 1924). Since these initial descriptions, numerous researchers have documented the presence of *Wolbachia* in a variety of somatic tissues (Table 1). In fact, the few examples in the literature where *Wolbachia* is restricted to the reproductive tissues, such as certain strains of the mosquito *Aedes albopictus* and female *Glossina morsitans* tsetse flies (Dobson et al., 1999), appear to be the exception rather than the rule. PCR and fluorescent cytological approaches have been used to assay for the presence of *Wolbachia,* with both techniques revealing a broad distribution in specific somatic cells and tissues (Table 1). Most of these data come from studies in either *Drosophila* or mosquitos. However, similar distribution patterns have been observed in numerous other insect and nematode species. Table 1 and Figure**** 1 depict the documented cellular and tissue distribution for these and other organisms.

**Table 1:** *Wolbachia* distribution in somatic tissues

Organism	Species	Somatic Tissues	References
Fruit Fly	*D. melanogaster* (adult)	Central brain (intra & extracellular), retina, optic lobe, ganglia, somatic cyst cells, somatic stem cells	Albertson et al., 2013; Casper‐Lindley et al., 2011; Strunov et al., 2013; Toomey et al., 2013; Veneti et al., 2003;
	*D. simulans* (adult)	Head, muscle, midgut, malpighian tubules, wings, hemolymph	Dobson et al., 1999; Osborne et al. 2009
	*D. melanogaster* (larva)	Nerves, malpighian tubules, salivary glands, trachea, fat body, proventriculus	Clark et al., 2005;
	*D. simulans* (larva)	Brain, salivary gland, midgut, fat body	Dobson et al., 1999;
Mosquito	*Ae. albopictus*	Salivary glands, some strains no somatic tissue	Dobson et al., 1999; Zouache et al., 2009;
	*An. gambiae (wMelPop)*	Brain, sensory organs, mouthparts, hemocytes, fat body, abdomen	Hughes, Koga et al., 2011;
	*C. pipiens*	Head, malpighian tubules, wings, hemolymph	Dobson et al., 1999;
	*C. cautella*	Head, muscles, midgut, malpighian tubules, wings, hemolymph	Dobson et al., 1999;
	*C. tarsalis*	Head, muscle, ganglia, fat body, ovary follicles	Dodson et al., 2014;
Nematode	*B. malayi*	Hypodermal chords, excretory canal, pseudocoelom	Fischer et al., 2011; Landmann et al., 2010;
	*M. perforate*	Epithelial gonad, intestinal wall	Ferri et al., 2011;
	*C. japonica*	Epithelial gonad	Ferri et al., 2011;
	*O. flexuosa*	Hypodermis, median chords, intestine	McNulty et al., 2013;
Tsetse Fly	*G. austeni*	Head, salivary gland, milk gland, fat body	Cheng et al., 2000;
	*G. brevipalpis*	No somatic tissue	Cheng et al., 2000;
	*G. morsitans*	No somatic tissue	Cheng et al., 2000;
Bed Bug	*C*. *lectularius*	Bacteriome, mesospermalage	Hosokawa et al., 2010;
Leafcutter Ant	*A*. *octospinosus*	Foregut, midgut, feces, muscle, thorax	Andersen et al., 2012;
Kissing Bug	*R. pallescens*	Salivary glands, intestine	Espino et al., 2009;
Termite	*C. subarquatus*	Head, salivary glands, thorax, legs	Roy et al., 2015

In brief, *Wolbachia* is prevalent in tissues of the nervous system in *Drosophila* and other flies (Albertson et al., 2013; Casper‐Lindley et al., 2011; Dobson et al., 1999; Mitsuhashi, Saiki, Wei, Kawakita, & Sato, 2002; Moreira et al., 2009; Osborne, Leong, O'Neill, & Johnson, 2009; Strunov & Kiseleva, 2014). In *Drosophila,* the distribution of the pathogenic *Wolbachia* strain, wMelPop, in the nervous system of adults is temperature dependent, with increased temperature favoring the expansion of *Wolbachia* from the central brain to peripheral areas such as the optic lobe and retina (Strunov, Kiseleva, & Gottlieb, 2013). These data suggest that temperature may be a possible determinant of *Wolbachia* replication in somatic tissues. In addition to the nervous system, *Wolbachia* is also present in digestive and metabolic tissues such as the fat body, gut, salivary glands, hemocytes, and malpighian tubules of various arthropod species where it may play a role in regulating host immunity and bioenergetics (Andersen, Boye, Nash, & Boomsma, 2012; Chevalier et al., 2011; Dobson et al., 1999; Faria & Sucena, 2013; Hughes, Koga, Xue, Fukatsu, & Rasgon, 2011; Hughes et al., 2011; Ponton et al., 2015; Zouache et al., 2009). *Wolbachia* has been further documented in muscle and wing tissue of some species (Andersen et al., 2012; Cheng et al., 2000; Dobson et al., 1999; Dodson et al., 2014; Frydman, Li, Robson, & Wieschaus, 2006; Min & Benzer, 1997), though the significance of this remains largely unknown. In filarial nematodes, the somatic distribution of *Wolbachia* is restricted to the lateral chords (hypodermis), excretory canal, and the intestine (Ferri et al., 2011; Fischer, Beatty, Jiang, Weil, & Fischer, 2011; Landmann et al., 2012).

The repeated observation of *Wolbachia* in specific somatic tissues suggests that somatic tissue tropism is not incidental, but rather a key aspect of *Wolbachia* biology. For instance, somatic localization of *Wolbachia* may be evolutionarily maintained because it aids horizontal transmission within and between species, thus serving as a mechanism to increase the genetic diversity of *Wolbachia*. Additionally, somatic *Wolbachia* may confer advantageous phenotypes in the host that enhance its germline transmission. Below, we further explore the mechanisms and functional significance of the somatic localization patterns of *Wolbachia*.

## 3 Segregation Patterns During oogenesis and Early Embryogenesis Influence Tissue Distribution Later in Development

### 3.1 Arthropods

As in many insect species, the *Drosophila* egg chamber consists of a syncytium of 15 nurse cells and an oocyte, all connected through cytoplasmic bridges (Spradling, 1993). During maturation, nurse cell cytoplasm is pumped into the oocyte. Importantly, specific determinants essential for anterior‐posterior (AP) axis formation are also transported from the nurse cells to the specific regions of the maturing oocyte. Localization of these AP axis and germline determinants requires microtubules, microtubule‐based motor proteins and association with posterior cortical cytoskeletal elements (Chang et al., 2011). Meanwhile, efficient transmission of most *Wolbachia* strains from one generation to the next requires that the bacteria concentrate at the posterior pole of the mature oocyte, as this is the future site of the germline (Kose & Karr, 1995). Thus, *Wolbachia* must migrate from the nurse cells to the posterior pole, navigating the constantly changing and tumultuous environment of the developing oocyte due to cytoplasmic streaming (Monteith et al., 2016). However, some strains, such as *Wolbachia* Riverside *(w*Ri*)* of *D. simulans* incorporate into the pole cells independently of posterior concentration by maintaining a high titer throughout the entire oocyte (Serbus & Sullivan, 2007; Veneti, Clark, Karr, Savakis, & Bourtzis, 2004), whereas others (*w*No*, w*Ma*, w*Ki) maintain a predominantly anterior localization (Veneti et al. 2004). These differences may ultimately contribute to differential somatic localization in adult flies.

Functional studies in *Drosophila* demonstrate that *Wolbachia* movement through the nurse cells to the anterior pole of the oocyte relies on the minus‐end directed motor protein dynein (Ferree et al., 2005). At this point in oogenesis the oocyte microtubules switch orientations such that transport to the posterior pole requires plus‐end directed microtubule movement. It has been difficult to attribute a functional significance of this dramatic switch in microtubule orientation, as well as cytoplasmic streaming. It may be that these are defense mechanisms preventing germline transmission of microbial invaders. Accordingly, *Wolbachia* rely on the plus‐end directed motor protein for transport and concentration at the posterior pole (Serbus & Sullivan, 2007). Finally, stable association with the posterior cortex requires key germ plasm and AP axis components such as Staufen and Oskar (Serbus & Sullivan, 2007). Thus, germline transmission of *Wolbachia* requires a sophisticated developmentally controlled association with dynein, kinesin, and finally conserved posterior determinants. Phylogenetic analyses of *Wolbachia* that vary in their niche tropism demonstrate that *Wolbachia*‐encoded factors are required for the posterior concentration (Toomey, Panaram, Fast, Beatty, & Frydman, 2013). One possibility is that *Wolbachia* expresses a developmentally programmed set of surface proteins that facilitates sequential engagement with host dynein, kinesin, and finally pole plasm determinants.

**Figure 2:**
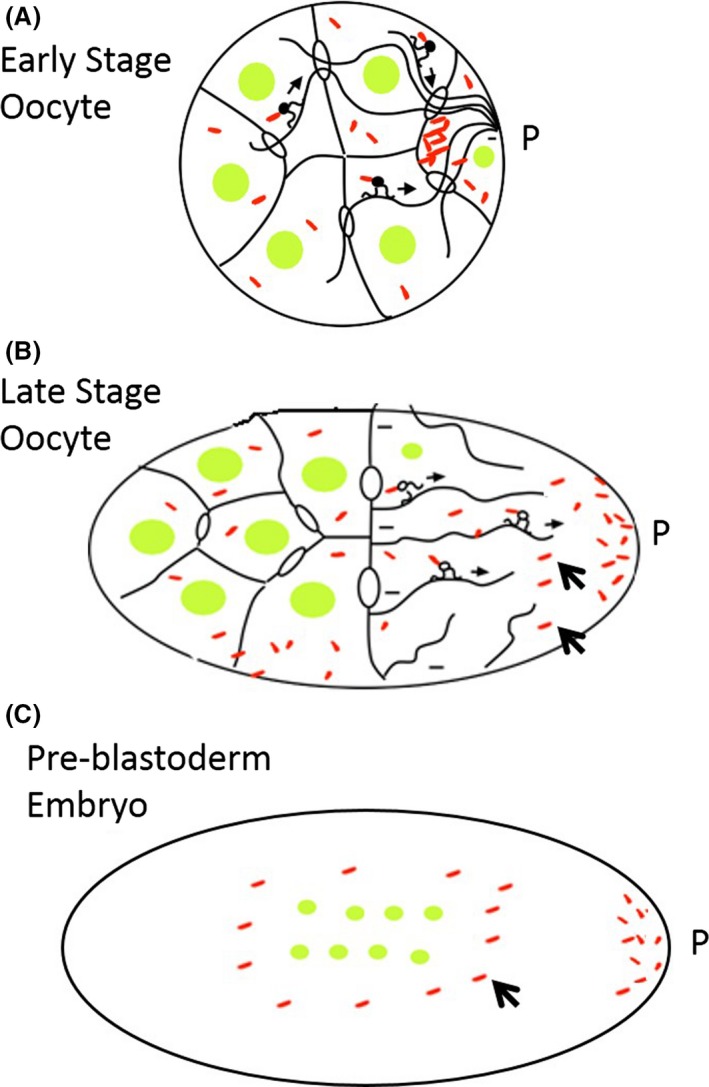
*Wolbachia* localization in somatic and germline cells during host development. The posterior localization of *Wolbachia* in the (A,B) developing oocyte and (C) embryo embryo relies on interactions with host microtubules, motor proteins, and posterior determinants. *Wolbachia* that localize to the posterior pole (P) are incorporated into the germline. However, a fraction of *Wolbachia* remains dispersed throughout the developing oocyte and embryo (arrowheads) and fate map to somatic tissues. Host nuclei=green, *Wolbachia=*red

In all insect species examined, there is also a significant fraction of *Wolbachia* that is not associated with the posterior cortex but remains dispersed throughout the oocyte, as shown in Figure 2 (Veneti et al., 2004). During the syncytial divisions following fertilization, these bacteria concentrate at the centrosomes and undergo cell‐cycle regulated movements along the spindle and astral microtubules associated with the dividing syncytial nuclei (Albertson, Casper‐Lindley, Jian, Tram, & Sullivan, 2009; Kose & Karr, 1995). As in the oocyte, it is likely this movement relies on the microtubule‐based motor proteins dynein and kinesin (Ferree et al., 2005). The functional significance of these movements is unclear. One possibility is that it serves to distribute *Wolbachia* throughout the embryo such that they will fate map to numerous developmental lineages. Thus, as with the oocyte, the final distribution of the *Wolbachia* throughout the cellularized embryo prior to gastrulation is determined by a combination of host and *Wolbachia* factors.

Examination of *Drosophila* larva reveals that, as embryonic development progresses, *Wolbachia* also concentrates in the embryonic and larval epithelial‐derived neuroblast stem cells (Albertson et al., 2009). In contrast to the symmetric segregation of *Wolbachia* in the syncytial divisions, *Wolbachia* in the neuronal lineage exhibits a highly asymmetric segregation pattern (Albertson et al., 2009). The dividing neuroblast produces a self‐renewing neuroblast daughter cell and a daughter cell that will differentiate into larval neurons. *Wolbachia* almost exclusively segregates with the neuroblasts with only a few bacteria localizing to the cells that will differentiate into larval neurons. This asymmetric localization and segregation is largely dependent on the robust astral microtubules associated with the self‐renewing neuroblast cell. Larval neuroblast cells undergo a period of quiescence and ultimately divide and differentiate into the cells that will become the adult central nervous system (Homem & Knoblich, 2012). Thus, the asymmetric neuroblast localization during the larval divisions ensures their eventual localization to the adult brain (Albertson et al., 2013).

Unfortunately, we know little about *Wolbachia* localization during the pupal stages. However, numerous studies that have examined its cellular and tissue distribution in the adult stages. These are described in section 2 (2) as well as Table 1 and Figure**** 1.

### 3.2 Filarial nematodes

As with arthropods, *Wolbachia* is inherited primarily through the female germline in filarial nematodes (Kozek, 1977). In insects, axis determination and the site of germline formation is established during oogenesis. In filarial nematodes an asymmetric MTOC is also present before fertilization, in contrast to the model nematode *Caenorhabditi*s *elegans*. Posterior localization of *Wolbachia* in both *Drosophila* and filarial nematodes relies on microtubules and motor proteins. During the establishment of polarity in the filarial nematode *Brugia malayi*,* Wolbachia* is associated with high levels of dynein, and dynein is required for their posterior localization (Landmann et al., 2014). Therefore, in both insects and filarial nematodes, microtubules and motor proteins are required for *Wolbachia* posterior enrichment. In addition, maintenance of *Wolbachia* at the posterior pole in *B. malayi* relies on posterior determinants, as in insects. The equivalence of the embryonic lineages between the model nematode *C. elegans* and *B. malayi* facilitates lineage tracing of *Wolbachia* in the latter. Such analysis revealed that during the initial embryonic divisions *Wolbachia* segregates with a precursor lineage to the germline and lateral chords (Caragata, Real, Zalucki, & McGraw, 2011; Fischer et al., 2011; Landmann, Foster, Slatko, & Sullivan, 2010). However, when this lineage diverges at the 12‐cell stage, *Wolbachia* segregates exclusively with the lateral chord lineages, leaving the germline lineage devoid of *Wolbachia*. This pattern of segregation is conserved in four filarial nematode species (*B. malayi, L. sigmondontis, D. immitis, O. japonica*), suggesting that it relies on conserved signaling factors associated with these species and perhaps others (Landmann et al., 2012). During the subsequent L3 and L4 larval stages, the hypodermal chords become syncytial through a process of cell fusion. Following this, *Wolbachia* proliferate extensively and spread anteriorly to fill the chord. In order to infect the germline, *Wolbachia* then migrate from the chord into the germline, crossing multiple plasma membranes. Images demonstrate that *Wolbachia* achieves this in female worms through the depolymerization of actin‐based microfilaments at the point of somatic‐germline cell contact (Landmann et al., 2012). Surprisingly, *Wolbachia* does not invade the germline in male nematodes, indicating *Wolbachia* is responding to signaling molecules specific to the female germline. Thus, in the late larva and adult males, *Wolbachia* is exclusively localized in the hypodermal lineage, whereas in females, *Wolbachia* resides in the hypodermal and germline lineages(Fischer et al., 2011; Landmann et al., 2012).

## 4 Molecular Mechanisms of Migration and Invasion of Somatic Cells

The studies described above indicate that in insects and nematodes the adult somatic distribution of *Wolbachia* is largely determined by a combination of symmetric and asymmetric segregation patterns during the mitotic divisions and cell‐to‐cell migration (Albertson et al., 2009; Landmann et al., 2010, 2012). With respect to the segregation patterns in both systems, it is clear that microtubules play a key role. Live imaging of the syncytial cortical divisions in *Drosophila* reveal *Wolbachia* maintains a tight association with the centrosome during interphase, but once the cell enters mitosis, *Wolbachia* undergoes extensive movement along pole to pole and astral microtubules (Albertson et al., 2009; Kose & Karr, 1995). Based on studies in the oocyte, this is likely to be driven by the microtubule‐based motor proteins dynein and kinesin (Ferree et al., 2005). This movement facilitates the even distribution of *Wolbachia* to daughter nuclei and serves to distribute them throughout the embryo. This is similar to what occurs in *B. malayi*, where *Wolbachia* moves along the astral and spindle microtubules during mitosis, facilitating their migration (Landmann et al., 2010). *Wolbachia* also relies on cortical microtubules and dynein to localize to the posterior cortex *in B. malayi* (Landmann et al., 2014).

The structural mechanisms by which *Wolbachia* engages host motor proteins and how this is regulated remain unknown. Sequence analysis reveals the *Wolbachia* genome contains several outer membrane proteins (WSPs, *Wolbachia* surface proteins) and these are likely to play a role in interacting with host cytoskeleton (Wu et al., 2004). However, electron microscopy has revealed that *Wolbachia* is encompassed by a host membrane (Callaini, Riparbelli, & Dallai, 1994; Fischer, Beatty, Weil, & Fischer, 2014), perhaps derived from the endoplasmic reticulum or golgi, making it difficult for WSPs to interact directly with the motor proteins. Nonetheless, some biochemical evidence indicates that WSPs directly bind host actin and *Wolbachia* interactions with host actin appear necessary for efficient migration of *Wolbachia* in the developing oocyte, as maternal transmission efficiency is greatly reduced in flies encoding cytoskeletal mutations (Melnikow et al., 2013; Newton, Savytskyy, & Sheehan, 2015). *Wolbachia* also encodes sec (Wu et al., 2004) and type IV secretion genes (Rances, Voronin, Tran‐Van, & Mavingui, 2008), suggesting the possibility that secreted effector proteins are used to interact with the host cytoskeleton. Notably, *Salmonella*, another intracellular bacterium that is also encompassed by a host membrane, utilizes an array of effector proteins to manipulate the cytoskeleton and achieve proper intracellular localization of the vacuole within which it resides. (LaRock, Chaudhary, & Miller, 2015).

Ultimately, interactions with host organelles, including the cytoskeleton, are used by intracellular bacteria in order to support replication and cell exit and entry. For instance, *Chlamydia* manipulates cytoskeletal Rab proteins in the host to recruit Golgi ministacks to the bacterial inclusion membrane in order to obtain lipids for cellular growth in human cells (Al‐Zeer et al., 2014; Heuer et al., 2009). Furthermore, *Neisseria* utilizes the host endocytic pathway for invasion through clathrin coated pits (Harvey, Jennings, Campbell, Williams, & Apicella, 2001). Since the ability of intracellular bacteria to manipulate host cells is in many cases conserved, the possibility that *Wolbachia* engage in similar processes to invade and persist in somatic cells should be further studied. For example*, Wolbachia* has been observed extracellularly in both the hemolymph of insects and pseudocoelomic cavity of filarial nematodes (Fischer et al., 2011, 2014). This localization indicates that *Wolbachia* is exocytosed and cell‐to‐cell transmission may occur through endocytosis. Accordingly, *Wolbachia* resides inside Golgi‐related vesicles near the host cell membrane in the *Drosophila* embryo (Cho, Kim, & Lee, 2011). In further support of an endocytosis hypothesis, free *Wolbachia* is able to invade uninfected germline tissues of *Anopheles* mosquitoes when the two are cocultured ex vivo (Hughes, Pike, Xue, & Rasgon, 2012). In these experiments, *Wolbachia* more efficiently invades tissues of their native hosts as opposed to those of more divergent ones. This suggests that *Wolbachia* enter cells through a receptor‐mediated mechanism that can be affected by polymorphisms in specific proteins that arise during speciation. These potential mechanisms are of particular importance to the finding that *Wolbachia* localizes to the somatic niche cells of the female germline in many *Drosophila* species (Fast et al., 2011; Toomey et al., 2013). Studies in which *Wolbachia* bacteria are injected into the adult abdomen demonstrate that *Wolbachia* can hone to these regions through migration (Frydman et al., 2006). How they achieve this remains unclear, as they must traverse a number of membrane and extracellular matrix barriers. However, receptor‐mediated endocytosis into specific cell types after movement through the hemolymph is a plausible route. Despite these intriguing lines of evidence, the role of the endocytic pathway in *Wolbachia* infection remains largely unexplored.

## 5 Horizontal Transmission of Infection

The discordance between *Wolbachia* and host phylogenies suggests that on evolutionary time scales horizontal transmission of *Wolbachia* between species has occurred numerous times. This conclusion is supported through studies demonstrating a strong linkage disequilibrium between mitochondrial and *Wolbachia* genomes in a number of species (Gómez‐Valero et al., 2004; Heath, Butcher, Whitfield, & Hubbard, 1999; Morrow, Frommer, Shearman, & Riegler, 2014; Schuler et al., 2013; Vavre, Fleury, Lepetit, Fouillet, & Boulétreau, 1999; Werren, Zhang, & Guo, 1995; Zhang, Han, & Hong, 2013). Such phylogenetic analyses provide clues to the most plausible routes of horizontal transmission. Horizontal transmission appears to take place within and between species through both direct and indirect interactions. For example, intraspecies horizontal transmission in organisms such as fruit flies and spiders likely happens through direct contact or the environment, given the ecological roles of these organisms do not allow for a vectored mechanism (Baldo et al., 2008; Haine, Pickup, & Cook, 2005). Likewise, interspecies horizontal transfer in intertidal amphipod crustaceans (Cordaux et al., 2001) and butterflies sharing the same habitat probably occurs through the environment (Dyson, Kamath, & Hurst, 2002). In plant‐feeding pumpkin arthropods, *Wolbachia* transfer appears to be linked to feeding on particular leaf substrates (Sintupachee, Milne, Poonchaisri, Baimai, & Kittayapong, 2006), suggesting that transfer can occur through ingestion. A similar link exists between predatory mirid bugs and their prey, leafhoppers (Kittayapong, Jamnongluk, Thipaksorn, Milne, & Sindhusake, 2003), whereas in mycophagous Diptera, the mushroom habitat appears to play a role in horizontal transmission (Stahlhut et al., 2010).

Whether horizontal transmission is a common occurrence on shorter time scales remains uncertain, though studies tracing *Wolbachia* movement among bee populations suggest it is an infrequent event (Gerth, Rothe, & Bleidorn, 2013). Analyses of cannibalistic terrestrial isopods have demonstrated new infections in various organs after ingestion of an infected individual by an uninfected one (Le Clec'h et al., 2013). Similarly, mixing experiments in the laboratory have shown that mites can transmit *Wolbachia* infection between *Drosophila* by feeding on infected corpses and subsequently being ingested by uninfected flies (Brown & Lloyd, 2015). In colonies of *Cubitermes* termites, the exchange of salivary secretions, also known as trophallaxys, appears to facilitate intraspecies transfer of *Wolbachia* between individuals of different castes (Roy, Girondot, & Harry, 2015). Thus, a similar route may be involved in other social insects. For example, in *Acromyrmex* ants, *Wolbachia* is present in the fat body, hemolymph, and feces, suggesting the potential for fecal‐oral transmission (Frost, Pollock, Smith, & Hughes, 2014). Interestingly, sequencing and FISH experiments have shown that parasitoid wasps are capable of horizontally acquiring new *Wolbachia* infections during larval development inside an infected host (Ahmed et al., 2015). Parasitoid wasps can also transmit their own vertically acquired *Wolbachia* to other coinfecting parasitoid species that may be occupying the same space during development inside a host (Huigens, de Almeida, Boons, Luck, & Stouthamer, 2004). In these examples, *Wolbachia* transmission is likely independent of the germline, relying solely on somatic tissues.

The mechanisms and routes of horizontal transmission are largely unexplored. However, some insight into these issues is provided by experimental transfer in the laboratory. Early experiments in *Drosophila* provided proof‐of‐principle that *Wolbachia* from one organism was capable of stably infecting another by localizing to the germline. That is, *Wolbachia* extracted from the cytoplasm of an infected *Drosophila* egg could be injected into an uninfected embryo and yield germline infection (Boyle, O'Neill, Robertson, & Karr, 1993). Experiments of a similar nature have since been conducted from adult to adult, and adult to immature stage insects of other species with varying degrees of success (Grenier et al., 1998; Kageyama, Narita, & Noda, 2008; Pigeault et al., 2014; Van Meer & Stouthamer, 1999). Though infection intensity appears to decline over time, in some cases stable germline infection can be achieved through injection (Grenier et al., 1998; Van Meer & Stouthamer, 1999). For example, *Wolbachia* injected into the abdomen of *Drosophila* can migrate to the germline (Frydman et al., 2006). Thus, one possible mechanism for natural horizontal transmission is through contact of an uninfected wounded individual with infected hemolymph from a wounded *Wolbachia* host, as has been demonstrated in woodlice (Rigaud & Juchault, 1995). Interestingly, experimental transfer of *Wolbachia* between related host species can in some cases be virulent and affect reproductive fitness (Le Clec'h et al., 2012; McGraw, Merritt, Droller, & O'Neill, 2002).

Given the diversity of interactions that appear to mediate horizontal transmission, it is likely that the phenomenon also occurs in other, yet undiscovered, *Wolbachia* hosts. It is particularly intriguing that a species barrier to horizontal transmission appears to exist, but that this can in some cases be overcome both in nature as described above, but also in the laboratory. For instance, the establishment of *Drosophila*‐derived *Wolbachia* infections in mosquito cell cultures has facilitated cross‐species transinfection in vivo (Dobson, Marsland, Veneti, Bourtzis, & O'Neill, 2002; McMeniman et al., 2008). It is not clear whether the species barrier is regulated by host or bacterial genes, as the molecular mechanisms governing horizontal transmission of infection have yet to be discovered and a variety of factors are possibly involved. Most prominently, the ability of *Wolbachia* to occupy and move through host somatic tissues such as the gut, and perhaps even the extracellular environment such as the hemolymph, are likely key components in horizontal transmission. This area remains relatively unexplored and future advances in understanding *Wolbachia* transit and invasion at the cellular level may yield greater understanding of the conditions required for horizontal transmission. In particular, studies that trace *Wolbachia* migration to somatic tissues after introduction through various routes are needed.

## 6 Extracellular Survival and Routes of Transmission

The ability of *Wolbachia* to transfer horizontally between organisms suggests that the bacterium is capable of surviving in an extracellular environment, though this idea is somewhat controversial. In the laboratory, *Wolbachia* has been isolated from both, infected cell cultures and tissues (Gamston & Rasgon, 2007; Rasgon, Gamston, & Ren, 2006). While *Wolbachia* obtained in this manner can be maintained in cell‐free medium and retain viability for at least a week, no replication is apparent. Nonetheless, these results indicate that *Wolbachia* is able to survive at least for a limited time outside of host cells. However, the fact that *Wolbachia* lack the ability to synthesize many essential lipids (Wu et al., 2004) and amino acids (Caragata, Rancès, O'Neill, & McGraw, 2014) is likely a major factor limiting the extent of extracellular survival.

Studies in vivo demonstrating the presence of *Wolbachia* in the hemolymph of both larvae and adults of *Drosophila* and mosquitoes provide further support for the idea that *Wolbachia* can survive extracellularly (Dobson et al., 1999; Frydman et al., 2006). Furthermore, when *Wolbachia* is injected into the abdomen of an uninfected *Drosophila* host, it is capable of surviving and migrating through the hemolymph to reach the germline (Frydman et al., 2006). From the hemolymph, *Wolbachia* may be able to also enter somatic tissues. For example, in the bedbug *Cimex lectularius, Wolbachia* is found in the mesospermalage, a hemocyte‐containing organ used to receive sperm during traumatic insemination (Hosokawa, Koga, Kikuchi, Meng, & Fukatsu, 2010). More importantly, contact with the infected hemolymph of wounded hosts can be a natural mechanism for horizontal transmission, as demonstrated by hemolymph transfer experiments (Rigaud & Juchault, 1995). Meanwhile, in the nematode *B. malayi*, extracellular *Wolbachia* are found in the pseuodocoelom, indicating that perhaps pseudoscoelomic fluid serves as a route for *Wolbachia* transfer between germline and somatic tissues (Fischer et al., 2014), similar to hemolymph in insects.

In addition to surviving in the hemolymph, *Wolbachia* has been observed extracellularly in various other important host tissues where it can exert both beneficial and harmful effects with respect to the host. For instance, while *Wolbachia* has been shown to concentrate in the central brain and optic lobe with little detriment (Albertson et al., 2013), studies show that some virulent *Wolbachia* strains can exit these cells, perhaps through cell lysis, and invade the extracellular space in the brain, causing pathogenesis (Min & Benzer, 1997; Strunov & Kiseleva, 2014).

A nutrient‐based symbiotic relationship may exist between extracellular *Wolbachia* and other hosts. For instance, in leaf‐cutter ants of the genus *Acromyrmex*,* Wolbachia* is observed extracellularly in the foregut, midgut lumen, and fecal fluid (Andersen et al., 2012; Frost et al., 2014; Sapountzis et al., 2015). *Wolbachia* is also found in the digestive tract of *Drosophila* (Clark, Anderson, Cande, & Karr, 2005; Ponton et al., 2015) and likely in triatomine bugs which excrete *Wolbachia* in their feces (Espino et al., 2009). These gut bacteria may provide essential metabolic pathways lacking from the insects, thereby controlling various aspects of host physiology and life history, and perhaps contributing to pathogen resistance.

Furthermore, in *C. lectularius*,* Wolbachia* resides within a highly specialized organ called the bacteriome (Hosokawa et al., 2010). The bacteriome is composed of bacteriocytes, a cell type similar to fat cells. These are maternally transmitted and serve primarily to protect endosymbiotic bacteria in exchange for nutrients. In this case, it appears *Wolbachia* may also be acting as a nutritional mutualist. Indeed, removal of endogenous *Wolbachia* from these bedbugs reduced host growth and reproductive fitness through a mechanism dependent on biotin synthesis (Nikoh et al., 2014).

Infection in extracellular compartments and the tissues discussed above may not only be important for horizontal transmission, but may also explain the various effects of *Wolbachia* on host physiology that appear to be independent of the germline. Across diverse taxa, the gut is a key tissue for regulating immunity, metabolism, and longevity. Likewise, the brain regulates these and other central processes while also controlling behavior. Thus, it is possible that the digestive tract is not only a route for *Wolbachia* transfer between hosts, but also, along with the brain, involved in the functional consequences of *Wolbachia* infection that are discussed below.

## 7 The Functions of Somatic Infection

In the mature oocyte, Wolbachia concentrates at the posterior pole facilitating its incorporation into the germline of the developing host embryo. In *Drosophila* and other insects, however, a large fraction of *Wolbachia* is also positioned anteriorly resulting in a distribution throughout the length of the embryos (Ferree et al., 2005; Serbus & Sullivan, 2007; Veneti et al., 2004). This *Wolbachia* fraction is not incorporated into the germline and fate maps to the somatic cells of the developing insect. In filarial nematodes, *Wolbachia* segregate to the posterior pole after fertilization and through asymmetric segregation all of the *Wolbachia* concentrate in the somatic hypodermal chords, leaving the germline uninfected. In females, a subset of these hypodermal *Wolbachia* invades the neighboring germline stem cells through cell‐to‐cell transfer. Strikingly in males invasion of the germline does not occur indicating this process relies on female germline‐specific signals (Landmann et al., 2010). Thus, unlike in insects where *Wolbachia* is distributed in most, if not all tissues of the adult, in filarial nematodes the only somatic tissue in which *Wolbachia* is consistently observed is the hypodermis.

In insects, the concentration of *Wolbachia* in the central nervous system, gut, and fat bodies, is particularly intriguing as these tissues direct many facets of insect behavior and physiology. The somatic distribution of *Wolbachia* may be viewed as a consequence of the fact that many *Wolbachia* fail to localize to the posterior pole and these bacteria are passively included into the newly formed somatic cells with little functional consequences. Alternatively, the dual somatic‐germline localization of *Wolbachia* may have evolved through positive selection in which somatically localized bacteria influence host cell biology and physiology such that vertical or horizontal transmission is enhanced. Below, we summarize evidence supporting the latter interpretation.

### 7.1 Effects on host behavior

There are many examples illustrating that vertically transmitted endosymbionts influence host behavior (Goodacre & Martin, 2012). Presumably these behavior modifications have evolved to enhance transmission of the endosymbiont. Over the past decade, a number of publications demonstrate that *Wolbachia* also has profound effects on insect behavior. This is likely a consequence of *Wolbachia* localization in the central nervous system and fat bodies, as they are hormone sources and influence physiology and behavior (Albertson et al., 2013; Arrese & Soulages, 2010; Nassel, 1993). A number of studies in *Drosophila* and spider mites have found that *Wolbachia* infection alters mating preference, duration, and frequency, as well as oviposition substrate preference (Goodacre & Martin, 2012; Koukou et al., 2006; Miller, Ehrman, & Schneider, 2010; Panteleev et al., 2007; Vala, Egas, Breeuwer, & Sabelis, 2004). However, a more recent study found no effect of *Wolbachia* infection on mating preference (Arbuthnott, Levin, & Promislow, 2016). Thus, the effect of Wolbachia on mating may be highly strain and host dependent.

In addition to mating behavior, feeding patterns appear to change during infection, as blood feeding success is reduced in *Wolbachia*‐infected mosquitoes (Turley, Moreira, O'Neill, & McGraw, 2009). While in this particular case, reduced feeding is not associated with reduced olfaction, other studies have found that *Wolbachia* can reduce host responsiveness to olfactory food cues (Peng, Nielsen, Cunningham, & McGraw, 2008). Changes in locomotor activity, also induced by *Wolbachia* infection in *Drosophila*, may contribute to apparent behavioral alterations (Caragata et al., 2011; Evans et al., 2009). While the mechanisms that underlie the phenomenon of behavioral change are undetermined, *Wolbachia* likely gain from altering essential host behaviors. Most prominently, changes in reproductive behavior may drive the spread of infection through populations by favoring the production of infected females. Similarly, changes in feeding behavior could confer a fitness advantage for infected individuals. For instance, in mosquitoes blood feeding is a costly behavior that can reduce fitness (Murdock, Moller‐Jacobs, & Thomas, 2013).

Many conclusions on the effects of *Wolbachia* on insect behavior must be treated with caution because the unaffected control insects are often obtained through antibiotic‐based curing of *Wolbachia*. Antibiotic treatment is certain to have profound effects on the composition of the gut and other host microbe populations (Broderick & Lemaitre, 2012). In addition, antibiotic treatment of *Drosophila* not infected with *Wolbachia* has dramatic long‐term effects on behavior and physiology, including mitochondrial function and lifespan (Albertson et al., 2013; Ballard & Melvin, 2007). Significantly, these effects persist many generations after the exposure to antibiotics (Albertson et al., 2013). Therefore, it is difficult to attribute the changes in behavior specifically to the loss of *Wolbachia*, despite the fact that most researchers attempt to control for this by curing several generations in advance of experimental manipulation. Given these issues, multiple generations of backcrossing is the preferred method of creating uninfected controls from infected insect lines when possible.

### 7.2 Effects on host metabolism

*Wolbachia* localization to the fat body, a key endocrine tissue in insects (Arrese & Soulages, 2010), has been observed on numerous occasions. The *Wolbachia* genome encodes an array of proteins that may be involved in regulating metabolism (Darby et al., 2012). This includes several facilitators of cation membrane transport that provide essential cofactors for enzymes in the respiratory chain. Furthermore, in filarial nematodes, *Wolbachia* can directly influence the expression of host enzymes involved in glucose and glycogen metabolism (Voronin et al., 2016). Therefore, it is unsurprising that *Wolbachia* increases the basal metabolic rate of infected mosquitoes as measured by the production of carbon dioxide (Evans et al., 2009). In *Drosophila*,* Wolbachia* also influence host iron‐utilization, whereas in *C. lectularius Wolbachia* appear to play a role in the synthesis of B vitamins (Brownlie et al., 2009; Hosokawa et al., 2010). These experiments suggest that *Wolbachia* not only affects macronutrient metabolism, but also the provisioning of mineral micronutrients and cofactors. In addition, some behavioral effects of *Wolbachia* in *Drosophila* may be explained by alterations in hormone biosynthesis pathways. For example, *w*MelPop may increase aggressive male behavior through control of octopamine synthesis (Rohrscheib et al., 2015). While these interesting effects on metabolism have not yet been explained, an increase in insulin signaling is one possible source of *Wolbachia'*s effects on host metabolism (Ikeya, Broughton, Alic, Grandison, & Partridge, 2009). Another possibility is that *Wolbachia* may affect mitochondrial mass or activity directly (Ballard & Melvin, 2007). Intriguingly, *Wolbachia*‐mediated metabolic alterations are suggestive of gainful manipulation of host physiology. Host diet in *Drosophila,* perhaps acting through the insulin signaling pathway, has been shown to regulate *Wolbachia* titer (Serbus et al., 2015). Therefore, it would not be surprising to discover that *Wolbachia,* like many other invasive bacteria, has the ability to modulate the metabolism of its host to increase its own transmission.

### 7.3 Cell autonomous and non‐autonomous effects on pathogen resistance

*Wolbachia* in infected flies and mosquitoes has the ability to confer resistance against a wide array of viral, bacterial, parasitic, and fungal pathogens (Eleftherianos et al., 2013). This property allows pathogen‐infected hosts to survive and continue to reproduce in a situation where uninfected hosts would not survive, thus providing a great evolutionary advantage for *Wolbachia* and its host. In mosquitoes, *Wolbachia* provides resistance against the malaria parasite *Plasmodium* (Kambris et al., 2010) and the filarial nematode *B*. *pahangi* (Kambris, Cook, Phuc, & Sinkins, 2009) as well as protection from the bacterium *Erwinia caratova* (Kambris et al., 2009) and the dengue and chikungunya viruses (Moreira et al., 2009). In *Drosophila*,* Wolbachia* infection imparts resistance against various positive‐sense single‐stranded RNA viruses such as: Drosophila C virus, noravirus, and cricket paralysis virus (Hedges, Brownlie, O'Neill, & Johnson, 2008; Rainey et al., 2016; Teixeira, Ferreira, & Ashburner, 2008) and against the entomopathogenic fungus *Beauveria bassiana* (Panteleev et al., 2007). However, *Wolbachia* protection does not include all infections. For instance, the titer of the intracellular bacteria *Salmonella typhimurium* and *Listeria monocytogenes* is not affected by *Wolbachia* in *Drosophila*, though it should be noted that these pathogens do not naturally infect flies (Rottschaefer & Lazzaro, 2012). Therefore, it is possible that *Wolbachia* may confer protection against intracellular bacteria that can naturally colozine arthropods.

Pathogen resistance imparted on the host by *Wolbachia* has been observed on numerous occasions and has been reviewed elsewhere (Eleftherianos et al., 2013). However, information regarding the conditions necessary for this phenotype, as well as mechanistic insight is still lacking (Rainey, Shah, Kohl, & Dietrich, 2014). One proposed mechanism is the priming of the immune response by *Wolbachia* that subsequently hastens pathogen removal upon infection. However, there is conflicting evidence for this claim and establishing a concrete link between *Wolbachia* and host immunity will greatly further understanding of the pathogen resistance phenotype (Bourtzis, Pettigrew, & O'Neill, 2000; Moreira et al., 2009; Rances et al., 2013; Wong, Hedges, Brownlie, & Johnson, 2011; Ye, Woolfit, Rances, O' Neill, & McGraw, 2013). Alternatively, some have suggested that the synthesis of reactive oxygen/nitrogen species and cholesterol is involved (Caragata et al., 2013; Pan et al., 2011; Wong, Brownlie, & Johnson, 2015). There is also some evidence that increased host cell autophagy driven by *Wolbachia* infection plays a role in viral resistance (Le Clec'h et al., 2012). Each of these mechanisms would require *Wolbachia*‐mediated effects on somatic tissues and cells that regulate the host response to infection, such as the gut, fat body, and hemocytes. The particular cells and tissues involved in each case are not fully known. In *Drosophila, Wolbachia* titer in the head, gut, and malpighian tubules is correlated with antiviral protection (Osborne et al., 2009). Furthermore, the emergence of fluorescence‐based assays for the detection of both *Wolbachia* and viruses have recently allowed for experiments that map their distribution and localization in whole insects (Kliot & Ghanim, 2015). In several tissues, such as the midgut and salivary glands, *Wolbachia* and dengue virus co‐localize. In such cases, it would appear that the effects of *Wolbachia* on dengue virus are cell autonomous, or restricted to the *Wolbachia‐*infected cells. However, viruses may also be impacted in a non‐autonomous manner due to *Wolbachia* in tissues where viruses are not present, such as Malpighian tubules and fat bodies that may control reactive oxygen species and cholesterol synthesis as mentioned above. Further studies of a similar nature should eventually facilitate greater understanding of the interactions between *Wolbachia* and pathogens in somatic cells.

### 7.4 Effects on stress resistance and longevity

As most mutualists and parasites, *Wolbachia* undoubtedly benefits from the health and longevity of its host. Therefore, it is not surprising that *Wolbachia* influences host responses to cellular stress and damage as well as lifespan. In insects, *Wolbachia* induces the production of host reactive oxygen species (ROS) (Pan et al. 2011; Wong et al., 2015). Perhaps because *Wolbachia* must persist in this oxidative intracellular environment without causing damage to the host, infection also upregulates host antioxidant genes (Brennan, Haukedal, Earle, Keddie, & Harris, 2012; Brennan, Keddie, Braig, & Harris, 2008). *Wolbachia* also reduces oxidative stress by regulating host iron homeostasis. Iron is a highly toxic precursor to ROS and the expression of *Wolbachia* bacterioferretin reduces labile iron concentrations, which in turn prevents toxicity (Kremer et al., 2009). Intriguingly, while *Wolbachia* protects against iron toxicity, resistance to lead is decreased during infection (Wang et al., 2012), suggesting that protection from heavy metals is restricted.

Reduced iron toxicity is associated with the inhibition of apoptosis in the wasp *Asobara tabida* (Kremer et al., 2009). In this organism, *Wolbachia* is required for proper oogenesis, and oocytes fail to mature when it is removed due to extensive apoptosis (Miller et al., 2010; Pannebakker et al., 2007). As mitochondria‐derived ROS are also involved in modulating apoptosis, the ability of *Wolbachia* to regulate responses to these stressors may have far reaching consequences for host lifespan and reproduction.

Whether *Wolbachia* modulates apoptosis from host germline or somatic tissues is unclear. Apoptosis in the wasp oocyte is likely due to *Wolbachia* in the same tissues. On the other hand, the loss of *Wolbachia* in filarial nematodes through antibiotic therapy also induces apoptosis in both the adult germline and somatic cells of the embryo (Landmann, Voronin, Sullivan, & Taylor, 2011). Since *Wolbachia* does not reside in the male germline of nematodes, this effect must be mediated by somatic *Wolbachia*. Significantly, apoptosis is upregulated in cells not infected with *Wolbachia* demonstrating that this effect is not cell autonomous. A greater understanding of the means by which *Wolbachia* regulate apoptosis is still necessary. Though some studies have suggested that *WSPs* are directly able to inhibit apoptosis (Bazzocchi et al., 2007), this mechanism does not account for apoptosis in tissues not infected with *Wolbachia*.

Ultimately the impact of *Wolbachia* on oxidative stress and apoptosis may affect host lifespan and longevity. For example, the removal of *Wolbachia* can decrease *Drosophila* lifespan (Alexandrov et al., 2007). Interestingly, a virulent strain of *Wolbachia* (*w*MelPop) in insect hosts can be pathogenic and induce apoptosis in a variety of tissues ultimately leading to death and reduced lifespan (Kambris et al., 2009; McMeniman et al., 2009; Min & Benzer, 1997; Strunov & Kiseleva, 2014; Zhukova & Kiseleva, 2012). Though such effects appear counterintuitive given *Wolbachia* gains from increased host fitness, perhaps pathogenicity and decreased lifespan contribute to the life history of *Wolbachia* in other ways.

### 7.5 Somatic routes of germline infection

The discordance between *Wolbachia* and host insect phylogenies strongly argues for multiple horizontal transmission events over evolutionary timescales. Insight into possible mechanisms and routes of transmission have come from experiments in which *Wolbachia* injected into the abdomen is able to reach the germline through the somatic stem cells (Frydman et al., 2006), suggesting that this localization during natural infection serves to facilitate reaching of the germline for vertical transmission. Indeed, from the somatic stem cell niche, *Wolbachia* is supplied to the somatic stem cell, which can then divide and transmit *Wolbachia* to follicle cells (Toomey et al., 2013). From infected follicle cells, *Wolbachia* may then transfer to the developing oocyte (Toomey et al., 2013).

Studies of oocytes isolated from wild caught *Drosophila* suggest that somatic to germline transmission of *Wolbachia* may be a common occurrence (Casper‐Lindley et al., 2011). Egg chambers isolated from infected females were discovered in which *Wolbachia* was absent from the early, but not the late stage chambers. These uninfected chambers are likely a consequence of an occasional failure of the *Wolbachia* in the germline stem cell to segregate during mitosis into the daughter cell that will become a nascent egg chamber. However, later in oogenesis, these chambers become infected. The most likely route of infection is from the *Wolbachia*‐infected follicle cells that encompass each egg chamber. Infection via these somatically derived follicle cells may have evolved as a backup mechanism to ensure the observed high rates of *Wolbachia* vertical transmission.

Perhaps the strongest support for a somatic to germline route of *Wolbachia* infection comes from the *Wolbachia* lineage studies in *B. malayi* described above in section 3. This analysis revealed that *Wolbachia* exclusively segregates to the lineage that forms the lateral chords. Here, it proliferates and completely fills the chords. At this point in larval development, no *Wolbachia* is present in the germline. Germline infection requires cell‐to‐cell transfer, or exiting the hypodermal chord cells and entering the adjacent germline cells. This mechanism of transfer remains unexplored, although cellular analysis suggests this involves *Wolbachia*‐mediated microfilament deploymerization at the point of entry.

## 8 Conclusions

While *Wolbachia* are most prevalent in the host germline and primarily studied for their effects on these tissues, the studies described in this review demonstrate that *Wolbachia* is consistently found both intra and extracellularly in important somatic tissues such as the nervous system, fat body, and gut of their arthropod hosts, and in hypodermal chords in the nematode hosts. *Wolbachia* distribution to these somatic tissues is primarily regulated by segregation patterns during embryonic development. However, active invasion of somatic tissues during development and adulthood is also involved. This mechanism not only regulates somatic distribution, but may be involved in the horizontal spread of infection, which appears to play an important ecological role in the transmission and diversification of *Wolbachia*. The presence of *Wolbachia* in somatic tissues may also explain many phenotypic alterations observed in infected hosts, such as: behavioral change, resistance to pathogenic infection, shifts in metabolism, and changes in longevity.

The effects that somatic *Wolbachia* has on the host germline suggest that invasion of the soma and somatic localization may have evolved as an altruistic mechanism to facilitate vertical transmission. That is, by not entering the germline, somatic *Wolbachia* are essentially sacrificed, as they will not be inherited by the next generation. However, in doing so, they can produce many of the phenotypes described above that increase the transmission of their sister *Wolbachia*, thus benefiting the species as a whole. Whether *Wolbachia* originated as a germline endosymbiont that invaded the soma resulting in these advantageous phenotypes or as a somatic endosymbiont that invaded the germline for vertical transmission remains unresolved. There are cases of *Wolbachia* existing exclusively in the germline (tsetse fly), but also exclusively in somatic tissues (male nematodes). In addition, invasion of both somatic and germline tissues has been documented, further obscuring the origins of *Wolbachia*.

Regardless of their origin, understanding the mechanisms by which somatic *Wolbachia* exert their effects has broad implications in the biomedical and agricultural fields, as the use of *Wolbachia* to manipulate the physiology of insect crop pests and vectors of human pathogens shows great potential to reduce disease and economic burden. However, studies examining *Wolbachia* invasion and interactions with host somatic cells at a mechanistic level are lacking. Some experiments suggest that *Wolbachia* manipulation of the host cytoskeleton and motor proteins plays an important role in cell invasion, but other aspects of host cell biology, such as the endocytic pathway may be involved as well. Thus, cell‐based studies of *Wolbachia* invasion that trace migration to specific somatic cells after introduction through various routes are sorely needed. Similarly, transmission studies focusing on transfer of *Wolbachia* between hosts under a variety of conditions will be helpful to fully determining the prevalent routes of horizontal transmission in nature. More importantly, studies directly mapping host phenotypes to *Wolbachia* in somatic tissues would greatly aid efforts to use this extraordinary endosymbiont for the public good.

## Funding Information

This study was funded by the National Institute of General Medical Sciences, (Grant / Award Number: ‘GM104486‘).

## Conflicts of Interest

The authors declare no conflicts of interest.
